# Career Decisions and In-Depth Insights Into Pediatric Ophthalmology as a Subspecialty Among Residents

**DOI:** 10.7759/cureus.84883

**Published:** 2025-05-27

**Authors:** Shahad F AlTayash, Rafaa Babgi, Seham Aljohar

**Affiliations:** 1 Department of Ophthalmology, King Khaled Eye Specialist Hospital, Riyadh, SAU; 2 Pediatric Ophthalmology Division, King Khaled Eye Specialist Hospital, Riyadh, SAU; 3 Epidemiology Section, Research Department, King Khaled Eye Specialist Hospital, Riyadh, SAU

**Keywords:** career, fellowship training, ophthalmology, pediatrics, residents, sub-specialty

## Abstract

Objective

This study explores ophthalmology residents’ awareness of various subspecialties, particularly pediatric ophthalmology and strabismus (POS), and examines the factors influencing their career decisions and overall satisfaction. It aims to identify barriers to pursuing POS and propose strategies to boost interest in this subspecialty in Saudi Arabia, where related research is limited.

Methodology

A cross-sectional study was conducted using purposive sampling. Data were collected through an electronic survey distributed to ophthalmology residents across various training programs in Saudi Arabia. The survey included sections on demographics, factors influencing career choices, and perceived barriers to selecting POS as a fellowship. Data were analyzed using IBM SPSS Statistics for Windows, Version 24.0 (Released 2016; IBM Corp., Armonk, NY, USA), employing descriptive statistics and bivariate analysis. A p-value of <0.05 was considered statistically significant.

Results

A total of 72 residents participated, most of whom were aged 25-30 and in their fourth year of residency. The most preferred subspecialties were anterior segment and surgical retina. Career decisions were significantly influenced by disease type and expected income. Notable gender differences were observed: male residents tended to prefer surgical retina, while female residents showed a greater preference for POS.

Conclusions

The findings highlight the need for targeted efforts to increase interest in pediatric ophthalmology and promote a more balanced distribution of specialists within the field.

## Introduction

Physicians in training make a series of decisions that guide them toward a specific area of medical practice. Ophthalmology offers a unique blend of primary and highly specialized care, encompassing both adult and pediatric populations and combining medical and surgical approaches. One key question every ophthalmology resident eventually faces is, “What subspecialty should I pursue?” Most residents enter their training already considering a specific subspecialty [1,2].

Numerous studies have explored the factors influencing physicians’ career choices. Most of these studies confirm that many medical professionals pursue ophthalmology to deepen their learning and ultimately specialize in a subspecialty rather than practice comprehensive ophthalmology alone [3-5]. These studies were prompted by concerns about physician workforce distribution, declining applicant numbers in certain subspecialties, changing gender demographics among medical graduates, and shifting priorities in career decision-making [3-5].

One such study by De Silva et al. [6] found that residents often aim to enter competitive fellowship programs, despite uncertainties regarding workforce demand. Studies by Gedde et al. [1], Ting et al. [3], and Al-Essa et al. [7] also revealed a growing number of residents intending to pursue fellowship training. Trends emerge with each new cohort of graduates, with certain subspecialties consistently ranking among residents' top choices due to their popularity and demand. Unfortunately, pediatric ophthalmology is not among them. Its popularity has declined, dropping from 9%, as reported by Alwadani et al. [8] to just 4%, raising concerns about the future of this field in Saudi Arabia [8].

This trend is not unique to Saudi Arabia. Hasan et al. [2] reported that only 7% of ophthalmology residents in the United States expressed interest in pediatric ophthalmology fellowship training. Choosing a fellowship is a significant decision that benefits from a better understanding of both career motivations and preferences, ultimately helping to align training opportunities with residents’ goals.

According to the World Health Organization, for every 10 million people, there should be at least one health facility with an ophthalmologist who is specifically trained or oriented in pediatric ophthalmology and strabismus (POS) [9]. Saudi Arabia’s Vision 2030 aligns with this recommendation. The government’s healthcare strategy includes enhancing physician training to strengthen the overall healthcare system [10]. To achieve this goal, the Ministry of Health has developed a Healthcare Transformation Strategy aimed at improving access to care by streamlining services across primary, secondary, and tertiary levels, reducing wait times, and boosting the quality of primary care [11].

The leading eye care institution in the country is King Khaled Eye Specialist Hospital (KKESH). Although major cities like Riyadh, the Eastern Region, and Jeddah have specialized eye hospitals, many areas remain underserved and lack adequate access to eye care. In 2012, the ophthalmologist-to-population ratio in Saudi Arabia was 1:43,000, and this demand is projected to rise in accordance with Vision 2030 [10,12-15].

Previous studies investigating interest in POS within the Saudi context have lacked multivariate controls or gender-based analyses, highlighting a gap in the literature that this study seeks to address.

Our objective is to explore the perceptions and attitudes of POS board-certified professionals in ophthalmology residency training: What drives their interest, and what barriers do they face in pursuing this subspecialty? Understanding these factors is essential to overcoming obstacles and fostering lasting interest in pediatric ophthalmology as a career path.

## Materials and methods

Study design and data collection

This cross-sectional study was conducted in 2023 using an online, self-administered questionnaire to evaluate career-choice decisions among Saudi ophthalmology residents. The survey was freely hosted on Google Forms (Google LLC, Mountain View, CA, USA) and distributed randomly via WhatsApp to all eligible Saudi ophthalmology residents. The inclusion criteria comprised residents in their second year of training or higher, as well as recent graduates.

The questionnaire explored both motivating and discouraging factors related to pursuing a career in pediatric ophthalmology.

Questionnaire variables

The questionnaire was specifically created for this study, based on its objectives and influenced by similar international research [2,8]. It was reviewed and validated by a pediatric ophthalmology specialist, and a pilot study was conducted to ensure the questions were clear and easy to understand. The questionnaire comprised 35 items exploring several aspects of the residents’ professional background and career motivations. These included demographic details such as age, gender, academic rank, and residency location, as well as factors that influenced their career decisions. Career factors assessed included diagnostic challenges, continuity of care, income, prestige, types of diseases encountered, on-call demands, research opportunities, and geographic location. These factors were measured using a five-point Likert scale ranging from 1 (strongly disagree) to 5 (strongly agree).

Participants were also asked to indicate their chosen ophthalmology subspecialty. To explore motivations for pursuing pediatric ophthalmology, the questionnaire addressed factors such as enjoyment of pediatric clinics, interest in strabismus, diversity of pediatric eye diseases, engaging research topics, enjoyment of working with children, rewarding clinical outcomes, variety of surgeries, and the perceived strength of the job market. Likewise, potential barriers were assessed, including difficulty dealing with anxious parents, challenges examining uncooperative children, lack of interest in the subject matter, insufficient training exposure, concerns about the job market and private income, and the necessity to cover various pediatric subspecialties. These responses were also rated using the five-point Likert scale.

Data analysis

The data were analyzed using IBM SPSS Statistics for Windows, Version 24.0 (Released 2016; IBM Corp., Armonk, NY, USA). Demographic, socioeconomic, and clinical characteristics were summarized accordingly. Due to limited responses in some subspecialty categories, Fisher’s exact test was used instead of the chi-square test when appropriate. Analyses were conducted based on age, gender, marital status, education level, and training location. An additional comparison was performed to examine gender-based preferences for pediatric ophthalmology, particularly among residents from KKESH.

Ethical considerations

Ethical approval for the study was obtained from the Research Ethics Board of KKESH in Riyadh, Saudi Arabia. Participation in the study was voluntary, and online informed consent was obtained from all respondents prior to completing the survey.

## Results

A total of 72 ophthalmology residents anonymously participated in the study. The majority of the respondents (n = 69, 95.8%) were between the ages of 25 and 30 years, while only three participants (4.2%) were aged 30 years or older, as shown in Table 1.

**Table 1:** Demographic characteristics of the respondents (n = 72) With chi-square tests as an addendum

Demographic characteristic		n (%)
Age	25-30	69 (95.8)
	>30	3 (4.2)
Gender	Male	37 (51.4)
	Female	35 (48.6)
Academic rank	R2	14 (19.4)
	R3	23 (31.9)
	R4	24 (33.3)
	Recently board-certified	11 (15.3)
Place of residency training	Riyadh (King Khaled Eye Specialist Hospital)	18 (25.0)
	Riyadh (King Saud University)	14 (19.4)
	Riyadh (National Guard Hospital)	2 (2.8)
	Riyadh (Prince Sultan Military Medical City)	4 (5.6)
	Eastern Region	19 (26.4)
	Western Region	9 (12.5)
	Madinah	3 (4.2)
	Assir	3 (4.2)

Of the residents who participated in the study, 37 (51.4%) were male and 35 (48.6%) were female. In terms of academic rank, most respondents were in their fourth year of residency (n = 24, 33.3%), followed by third-year residents (n = 23, 31.9%), second-year residents (n = 14, 19.4%), and recently board-certified ophthalmologists (n = 11, 15.3%).

Among the training centers across Saudi Arabia, the Eastern Region had the highest number of participating residents (n = 19, 26.4%), followed by KKESH, Riyadh (n = 18, 25%), and King Saud University, Riyadh (n = 14, 19.4%). The Western Region contributed nine participants (12.5%), while Prince Sultan Military Medical City, Riyadh, had four participants (5.6%). Both the Madinah and Asir regions had an equal number of respondents (n = 3, 4.2%), and the National Guard Hospital, Riyadh, had the fewest participants (n = 2, 2.8%).

Table 2, Table 3, and Table 4 serve as adjuncts to Table 1 and present the results of chi-square analyses comparing various factors in relation to gender.

**Table 2:** Chi-square test: age * gender ^a^ Two cells (50.0%) have expected counts less than 5. The minimum expected count is 1.46. ^b^ Computed only for a 2 × 2 table. ^c^ The standardized statistic is -1.709.

Age * gender	Value	df	Asymptotic significance (two sided)	Exact significance (2-sided)	Exact significance(one sided)	Point probability
Pearson chi-square	2.961^a^	1	0.085	0.240	0.130	
Continuity correction^b^	1.279	1	0.258			
Likelihood ratio	4.118	1	0.042	0.240	0.130	
Fisher’s exact test				0.240	0.130	
Linear-by-linear association	2.920^c^	1	0.087	0.240	0.130	0.130
Number of valid cases	72					

**Table 3:** Chi-square test: academic rank * gender ^a^ No cells (0.0%) have expected counts less than 5. The minimum expected count is 5.35. ^b^ The standardized statistic is -0.375.

Academic rank * gender	Value	df	Asymptotic significance (two sided)	Exact significance (two sided)	Exact significance (one sided)	Point probability
Pearson chi-square	3.216^a^	3	0.360	0.373		
Likelihood ratio	3.303	3	0.347	0.373		
Fisher-Freeman-Halton exact test	3.158			0.376		
Linear-by-linear association	0.141^b^	1	0.707	0.720	0.400	0.089
Number of valid cases	72					

**Table 4:** Chi-square test: place of residency training * gender ^a ^Ten cells (62.5%) have expected counts less than 5. The minimum expected count is 0.97. ^b ^The standardized statistic is 0.499.

Place of residency training * gender	Value	df	Asymptotic significance (two sided)	Exact significance (two sided)	Exact significance (one sided)	Point probability
Pearson chi-square	12.079^a^	7	0.098	0.081		
Likelihood ratio	15.585	7	0.029	0.056		
Fisher-Freeman-Halton exact test	11.095			0.096		
Linear-by-linear association	0.249^b^	1	0.618	0.630	0.329	0.038
Number of valid cases	72					

**Figure 1:**
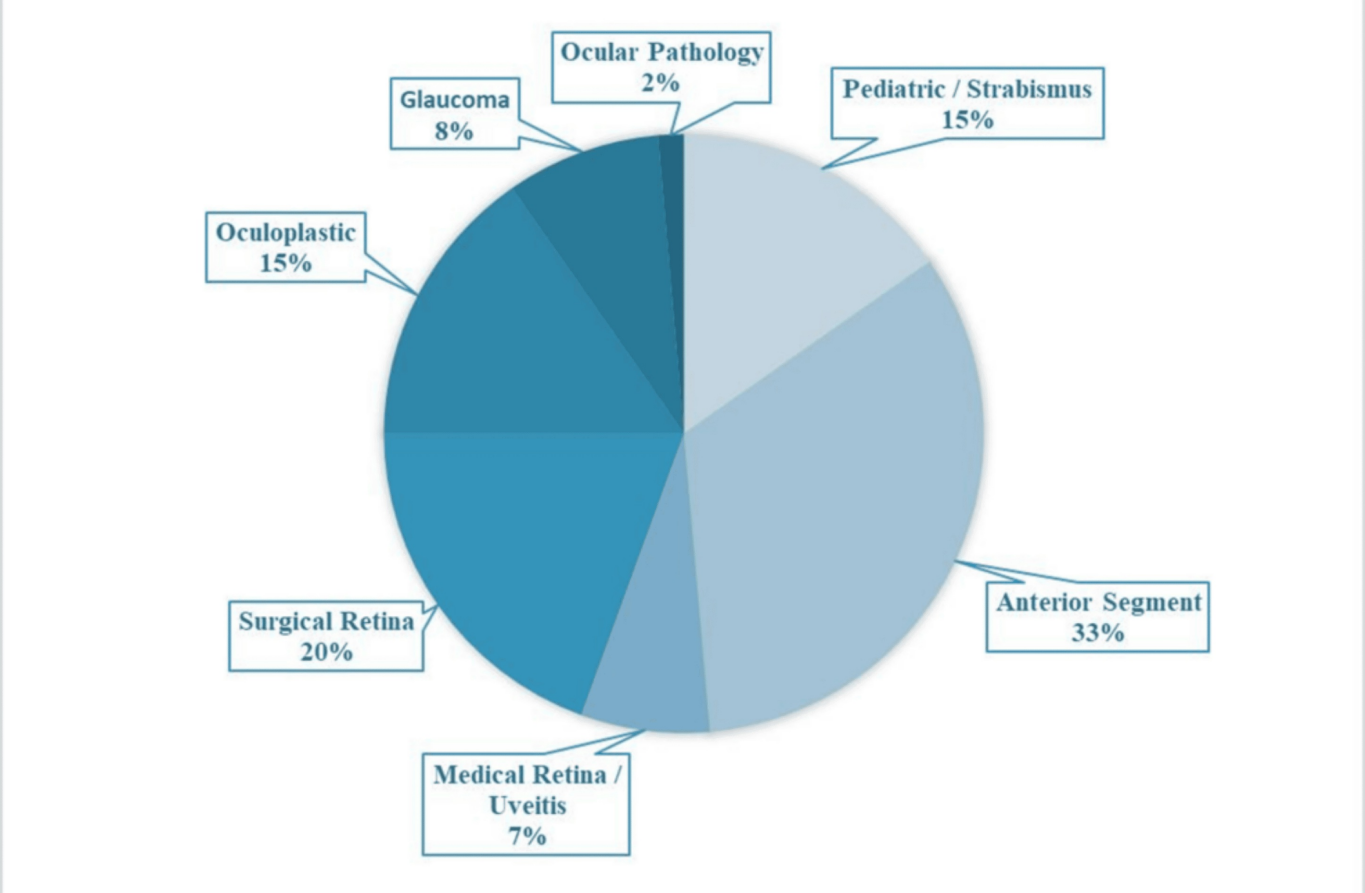
Future subspecialty/career choice among respondents (n = 72)

Figure 1 shows that the anterior segment is the most popular subspecialty among residents, with 24 participants (33%) selecting it as their preferred career path. Surgical retina follows as the second most favored option with 14 residents (20%). Pediatrics/strabismus and oculoplastics are tied in popularity, each chosen by 11 residents (15%). In contrast, medical retina/uveitis and glaucoma are less frequently selected, with five (7%) and six (8%) residents choosing them, respectively. Subspecialties such as neuro-ophthalmology and ocular genetics received no interest at all, while ocular pathology was the least popular, selected by only one participant (2%). These findings highlight a strong preference for anterior segment and surgical retina training, with markedly low interest in several other subspecialties.

Table 5 presents data on the factors influencing career choice, based on responses to scale questions rated from 1 to 5, where 1 = Strongly disagree/Not at all, and 5 = Strongly agree/Very much.

**Table 5:** Factors influencing career choice scale questions 1 = Strongly disagree/Not at all; 5 = Strongly agree/Very much

Factor	1, n (%)	2, n (%)	3, n (%)	4, n (%)	5, n (%)
Challenging diagnostic problems	5 (6.9)	14 (19.4)	23 (31.9)	23 (31.9)	7 (9.7)
Continuity of care	7 (9.1)	13 (18.1)	19 (26.4)	26 (36.1)	7 (9.7)
Income	3 (4.2)	9 (12.5)	17 (23.6)	25 (34.7)	18 (25.0)
Prestige	9 (12.5)	19 (26.4)	21 (29.2)	19 (26.4)	4 (5.6)
Type of diseases	2 (2.8)	5 (6.9)	13 (18.1)	16 (22.2)	36 (50.0)
On calls	5 (6.9)	7 (9.7)	11 (15.3)	21 (29.2)	28 (38.9)
Research opportunities	15 (20.8)	13 (18.1)	20 (27.8)	10 (13.9)	14 (19.4)
Geographic location	13 (18.1)	7 (9.7)	13 (18.1)	20 (27.8)	19 (26.4)

For the factor challenging diagnostic problems, 31.9% of respondents (n = 23) rated its influence as moderate (3) or agree (4), with an even split between these two ratings. Most respondents (n = 23; 31.9%) considered it somewhat influential (rating: 3). In contrast, the factor continuity of care received higher influence ratings, with 36.1% (n = 26) rating it as influential (4) and 9.7% (n = 7) as very influential (5).

Regarding income, a substantial number of respondents indicated strong influence, with 34.7% (n = 25) rating it as influential (4) and 25.0% (n = 18) rating it very influential (5). The factor prestige showed a more mixed response: 29.2% (n = 21) rated it as moderate (3), while smaller percentages assigned high (4) or very high (5) influence.

The factor type of diseases stands out, with 50.0% (n = 36) rating it as very influential (5), indicating it as the most strongly favored factor. Similarly, on-calls were also seen as highly influential, with 38.9% (n = 28) rating it very influential (5) and 29.2% (n = 21) rating it influential (4).

Research opportunities showed a broader range of responses, with 27.8% (n = 20) rating it moderately influential (3), but 20.8% (n = 15) strongly disagreeing (1). Lastly, geographic location demonstrated a balanced distribution across all ratings, especially moderate (3) and high (4) influences.

Overall, Table 5 highlights that the type of diseases and on-call duties are perceived as having the strongest impact on career choice, whereas research opportunities show more varied influence.

Table 6 presents data on factors influencing residents interested in pediatric ophthalmology, also rated on a scale from 1 (strongly disagree/not at all) to 5 (strongly agree/very much). Each factor shows a distinct response pattern, offering insight into the diverse motivations and levels of interest among residents.

**Table 6:** Factors influencing residents’ decision to consider pediatric ophthalmology as a future subspecialty (n = 72) 1 = Strongly disagree/Not at all, and 5 = Strongly agree/Very much

Factor	1, n (%)	2, n (%)	3, n (%)	4, n (%)	5, n (%)
I enjoy the pediatric clinic	23 (31.9)	14 (19.4)	14 (19.4)	13 (18.1)	8 (11.1)
I like strabismus	20 (27.8)	10 (13.9)	14 (19.4)	14 (19.4)	14 (19.4)
Varieties of diseases	18 (25.0)	16 (22.2)	16 (22.2)	16 (22.2)	6 (8.3)
Research is very interesting in this field	23 (31.9)	16 (22.2)	23 (31.9)	6 (8.3)	4 (5.6)
I enjoy dealing with the pediatric age group	33 (45.8)	8 (11.1)	11 (15.3)	13 (18.1)	7 (9.7)
Usually, the outcome is rewarding	15 (20.8)	7 (9.7)	16 (22.2)	20 (27.8)	14 (19.4)
Different types of surgeries	17 (23.6)	6 (8.3)	15 (20.8)	18 (25.0)	16 (22.2)
Has a good job market	17 (23.6)	8 (11.1)	16 (22.2)	14 (19.4)	17 (23.6)
Difficulty dealing with anxious parents	14 (19.4)	6 (8.3)	14 (19.4)	23 (31.9)	15 (20.8)
Difficulty examining an uncooperative child	7 (9.7)	6 (8.3)	4 (5.6)	19 (26.4)	36 (50.0)
No interest in the subjects of pediatric ophthalmology	21 (29.2)	10 (13.9)	15 (20.8)	7 (9.7)	19 (26.4)
Lack of exposure during my training	36 (50.0)	14 (19.4)	10 (13.9)	7 (9.7)	5 (6.9)
Poor job market/private income	22 (30.6)	14 (19.4)	21 (29.2)	9 (12.5)	6 (8.3)
Covering different subspecialties in pediatric age groups	21 (29.2)	20 (27.8)	13 (18.1)	12 (16.7)	6 (8.3)

For the factor “I enjoy the pediatric clinic,” a notable portion of residents (n = 21, 31.9%) strongly disagree, indicating low interest, while only a small group (n = 8, 11.1%) strongly agree. Similarly, the factor “I like strabismus” shows a more balanced distribution, with 19.4% of residents spread fairly evenly across the higher interest levels (3, 4, and 5). For the factor “Varieties of diseases,” there is a relatively high percentage of residents (n = 18, 25.0%) who strongly disagree, but also a significant portion (n = 16, 22.2%) showing moderate to high interest (levels 3 and 4).

The factor “Research is very interesting in this field” reveals that a substantial number of residents (n = 23, 31.9%) strongly disagree, an equal percentage remain neutral, and only a small number (n = 4, 5.6%) strongly agree. The factor “I enjoy dealing with the pediatric age group” stands out with the highest percentage of strong disagreement (n = 33, 45.8%), suggesting a considerable lack of interest in this aspect of pediatric ophthalmology. Conversely, the factor “usually, the outcome is rewarding” shows a more positive trend, with 27.8% (n = 20) agreeing (level 4) and 19.4% (n = 14) strongly agreeing.

Factors such as “Different types of surgeries” and “Has a good job market” both show higher levels of interest. For surgeries, 25.0% (n = 18) of residents agree at level 4, and 22.2% (n = 16) strongly agree. Similarly, for the job market, 19.4% (n = 14) agree at level 4, and 23.6% (n = 17) strongly agree. These findings suggest that certain motivations for pursuing pediatric ophthalmology resonate more strongly with the residents, reflecting diverse but specific interests within this group.

In contrast, Table 7 outlines the factors that discourage residents from choosing pediatric ophthalmology as a subspecialty.

**Table 7:** Factors influencing residents interested in pediatric ophthalmology as a future subspecialty (n = 11) 1 = Strongly disagree/Not at all, and 5 = Strongly agree/Very much

Factor	1, n (%)	2, n (%)	3, n (%)	4, n (%)	5, n (%)
I enjoy the pediatric clinic	0 (0.0)	2 (18.2)	0 (0.0)	4 (36.4)	5 (45.5)
I like strabismus	0 (0.0)	1 (9.1)	2 (18.2)	4 (36.4)	4 (36.4)
Varieties of diseases	1 (9.1)	2 (18.2)	1 (9.1)	6 (54.5)	1 (9.1)
Research is very interesting in this field	1 (9.1)	4 (36.4)	5 (45.5)	1 (9.1)	0 (0.0)
I enjoy dealing with the pediatric age group	1 (9.1)	2 (18.2)	1 (9.1)	4 (36.4)	3 (27.3)
Usually, the outcome is rewarding	0 (0.0)	2 (18.2)	2 (18.2)	3 (27.3)	4 (36.4)
Different types of surgeries	1 (9.1)	2 (18.2)	1 (9.1)	5 (45.5)	2 (18.2)
Has a good job market	1 (9.1)	0 (0.0)	5 (45.5)	2 (18.2)	3 (27.3)

A significant portion of residents express strong enthusiasm for pediatric clinics, with 45.5% (n = 5) strongly agreeing and 36.4% (n = 4) agreeing. Only a small percentage (18.2%, n = 2) rated this factor lower, indicating overall positive feelings toward working in pediatric clinics among these residents. Similarly, a strong liking for strabismus is evident, with 36.4% (n = 4) rating it a 5 and another 36.4% (n = 4) rating it a 4, showing that the majority of residents are passionate about pediatric ophthalmology, as reflected in Table 8.

**Table 8:** Reasons for residents’ disinterest in pediatric ophthalmology 1 = Strongly disagree/Not at all, and 5 = Strongly agree/Very much

Factor	1, n (%)	2, n (%)	3, n (%)	4, n (%)	5, n (%)
Difficulty dealing with anxious parents	14 (19.4)	6 (8.3)	14 (19.4)	23 (31.9)	15 (20.8)
Difficulty examining an uncooperative child	7 (9.7)	6 (8.3)	4 (5.6)	19 (26.4)	36 (50.0)
No interest in the subjects of pediatric ophthalmology	21 (29.2)	10 (13.9)	15 (20.8)	7 (9.7)	19 (26.4)
Lack of exposure during my training	36 (50.0)	14 (19.4)	10 (13.9)	7 (9.7)	5 (6.9)
Poor job market/private income	22 (30.6)	14 (19.4)	21 (29.2)	9 (12.5)	6 (8.3)
Covering different subspecialties in pediatric age groups	21 (29.2)	20 (27.8)	13 (18.1)	12 (16.7)	6 (8.3)

Regarding the variety of diseases, more than half of the residents (n = 6, 54.5%) rated this factor as “agree” (4), and one resident (n = 1, 9.1%) rated it as “strongly agree” (5), reflecting a generally positive attitude. However, a smaller group (n = 2, 18.2%) rated it lower, showing some variation in interest. Interest in research within pediatric ophthalmology showed a more mixed pattern: 45.5% (n = 5) rated it as neutral (3), while a notable number (n = 4, 36.4%) rated it as somewhat disagree (2), suggesting research is not a primary motivating factor for many residents.

Enjoyment of working with the pediatric age group was another important factor, with 36.4% (n = 4) agreeing and 27.3% strongly agreeing. Still, some residents (n = 2, 18.2%) showed less enthusiasm, rating it a 2, indicating that while many enjoy this aspect, others are less drawn to it. The belief that the outcomes in pediatric ophthalmology are rewarding was widely shared, with 36.4% (n = 4) strongly agreeing and 27.3% (n = 3) agreeing, though a few residents remained neutral.

The variety of surgical procedures available was viewed positively by most residents, with 45.5% (n = 5) agreeing and 18.2% (n = 2) strongly agreeing that this was an important factor. A minority were less favorable. Similarly, the perception of a good job market was mostly positive, with 27.3% (n = 3) strongly agreeing and 45.5% (n = 5) rating it as neutral (3), though opinions varied.

For the factor concerning difficulty dealing with anxious parents, 31.9% (n = 23) of residents showed moderate agreement (level 4), and 20.8% (n = 15) strongly agreed that this was a deterrent, indicating a significant level of disinterest related to this challenge. Conversely, 19.4% (n = 14) strongly disagreed, showing fewer residents were firmly opposed to this as a barrier. Regarding difficulty examining uncooperative children, half of the residents (n = 36, 50.0%) strongly agreed that this influenced their disinterest, with an additional 26.4% (n = 19) agreeing moderately. This highlights a key reason for avoiding this subspecialty, as shown in Table 8.

The factor “No interest in the subjects of pediatric ophthalmology” shows a varied distribution, with 29.2% of residents (n = 21) strongly disagreeing, indicating that this reason is not a major factor for many. However, 26.4% (n = 19) strongly agree, reflecting a notable segment who find this reason significant for their disinterest. The factor “Lack of exposure during my training” stands out, with half of the residents (n = 36, 50.0%) strongly disagreeing, suggesting this is not widely seen as a cause of disinterest, while only 6.9% (n = 5) firmly agree. Regarding the factor “Poor job market/private income,” opinions are mixed: 30.6% (n = 22) strongly disagree, and 29.2% (n = 21) remain neutral, with only 8.3% (n = 6) strongly agreeing, indicating limited conviction in this reason. The factor “Covering different subspecialties in pediatric age” also receives mixed responses, with 29.2% (n = 21) strongly disagreeing and 27.8% (n = 20) somewhat disagreeing, while only 8.3% (n = 6) strongly agree, showing a general lack of consensus on this factor as a cause for disinterest.

Overall, these data reflect a range of reasons for residents’ disinterest in pediatric ophthalmology, with some factors resonating more strongly than others. Notably, “Difficulty dealing with anxious parents” and “lack of exposure during my training” show contrasting high levels of agreement and disagreement, respectively, highlighting clear influences on residents’ attitudes.

When comparing male and female responses in Table 9 (n = 72) across various factors, both age groups (25-30 and over 30) display similar distributions by gender, with no significant differences (p = 0.240). The distribution of participants across residency ranks R2, R3, and R4 is also relatively even, showing no significant gender differences (p = 0.376). However, a higher percentage of males (11.1%) are recently board-certified compared to females (4.2%).

**Table 9:** Comparison of responses between male and female participants (n = 72) Fisher’s exact test of association (P < 0.05) * Independent samples t-test (P < 0.05)

Factor		Male, n (%)	Female, n (%)	P
Age	25-30	34 (47.2)	35 (48.6)	0.24
	>30	3 (4.2)	0 (0.0)	
Academic rank	R2	8 (11.1)	6 (8.3)	0.376
	R3	11 (15.3)	12 (16.7)	
	R4	10 (13.9)	14 (19.4)	
	Recently board-certified	8 (11.1)	3 (4.2)	
Place of residency training	Riyadh (King Khaled Eye Specialist Hospital)	8 (11.1)	10 (13.9)	0.096
	Riyadh (King Saud University)	9 (12.5)	5 (6.9)	
	Riyadh (National Guard Hospital)	2 (2.8)	0 (0.0)	
	Riyadh (Prince Sultan Military Medical City)	0 (0.0)	4 (5.6)	
	Eastern Region	12 (16.7)	7 (9.7)	
	Western Region	5 (6.9)	4 (5.6)	
	Madinah	0 (0.0)	3 (4.2)	
	Assir	1 (1.4)	2 (2.8)	
Subspecialty of choice	Pediatric/strabismus	2 (2.8)	9 (12.5)	0.004
	Anterior segment	11 (15.3)	13 (18.1)	
	Medical retina/uveitis	3 (4.2)	2 (2.8)	
	Surgical retina	13 (18.1)	1 (1.4)	
	Oculoplastic	5 (6.9)	6 (8.3)	
	Neuro-ophthalmology	0 (0.0)	0 (0.0)	
	Glaucoma	3 (4.2)	3 (4.2)	
	Ocular pathology	0 (0.0)	1 (1.4)	
	Ocular genetics	0 (0.0)	0 (0.0)	
* Factors influencing final career choice (mean Likert score ± SD)	Challenging diagnostic problems	3.35 ± 1.03	3.00 ± 1.11	0.169
	Continuity of care	3.27 ± 1.17	3.09 ± 1.12	0.497
	Income	3.92 ± 1.06	3.34 ± 1.11	0.028
	Prestige	3.24 ± 1.06	2.46 ± 1.04	0.002
	Type of diseases	4.14 ± 1.06	4.06 ± 1.16	0.767
	On calls	3.78 ± 1.29	3.89 ± 1.21	0.731
	Research opportunities	3.11 ± 1.45	2.74 ± 1.34	0.271
	Geographic location	3.43 ± 1.56	3.26 ± 1.31	0.608

When examining academic rank across different training centers, slight differences emerge in participant distribution. The Eastern Region has the highest number of participants, especially among males (16.7%), although these differences are not statistically significant (p = 0.096). Regarding subspecialty preferences, significant gender differences are evident. More females prefer pediatric/strabismus (n = 9, 12.5%) compared to males (n = 2, 2.8%) (p = 0.004), while males show greater interest in surgical retina (n = 13, 18.1%) than females (n = 1, 1.4%).

The table also compares mean Likert scores for factors influencing career choice by gender. For challenging diagnostic problems, males have a slightly higher mean score (3.35) than females (3.00), but this difference is not significant (p = 0.169). Similarly, mean scores for continuity of care are comparable between males (3.27) and females (3.09), with no significant difference (p = 0.497).

Males report significantly higher mean scores for income (3.92 vs. 3.34, p = 0.028) and prestige (3.24 vs. 2.46, p = 0.002). No significant gender differences are observed for factors such as type of diseases, on-call duties, research opportunities, or geographic location.

Table 10 further details comparisons of male and female KKESH residents’ responses across various factors. Age distribution is fairly balanced, with 44.4% of males (n = 8) and 55.6% of females (n = 10) aged 25-30, indicating a slight predominance of younger females in the group.

**Table 10:** Comparison of male and female King Khaled Eye Specialist Hospital residents’ responses (n = 18) Fisher’s exact test of association (P < 0.05)

Factor		Male, n (%)	Female, n (%)	P
Age	25-30	8 (44.4)	10 (55.6)	
	>30	0 (0.0)	0 (0.0)	
Academic rank	R2	1 (5.6)	3 (16.7)	0.835
	R3	3 (16.7)	3 (16.7)	
	R4	3 (16.7)	3 (16.7)	
	Recently board-certified	1 (5.6)	1 (5.6)	
Subspecialty of choice	Pediatric/strabismus	0 (0.0)	3 (16.7)	0.016
	Anterior segment	2 (11.1)	2 (11.1)	
	Medical retina/uveitis	0 (0.0)	1 (5.6)	
	Surgical retina	6 (33.3)	1 (5.6)	
	Oculoplastic	0 (0.0)	3 (16.7)	
	Neuro-ophthalmology	0 (0.0)	0 (0.0)	
	Glaucoma	0 (0.0)	0 (0.0)	
	Ocular pathology	0 (0.0)	0 (0.0)	
	Ocular genetics	0 (0.0)	0 (0.0)	

Regarding academic rank, the distribution across R2, R3, and R4 levels, as well as recently board-certified residents, is quite similar between male and female participants, with no significant differences observed (p = 0.835). This suggests that both genders are advancing through their residency programs at comparable rates.

Significant gender differences arise in subspecialty preferences. Specifically, 16.7% of female residents (n = 3) expressed interest in pediatric/strabismus, while no male residents chose this subspecialty. Conversely, surgical retina was preferred by 33.3% of male residents (n = 6) compared to only 5.6% of females (n = 1). These differences are statistically significant (p = 0.016), highlighting distinct gender-based trends in career specialization among KKESH residents.

When examining factors influencing final career choices (Table 11), males rated challenging diagnostic problems slightly higher, with a mean score of 3.25 compared to 2.60 for females; however, this difference was not statistically significant (p = 0.214). Similarly, both genders gave comparable ratings for continuity of care, with mean scores of 3.00 for males and 2.80 for females, showing no significant difference (p = 0.775).

**Table 11:** Comparison of male and female King Khaled Eye Specialist Hospital residents’ preferences influencing career choice (n = 18) Independent samples t-test (P < 0.05)

Factor		Male (mean ± SD)	Female (mean ± SD)	P
Factors influencing final career choice	Challenging diagnostic problems	3.25 ± 1.28	2.60 ± 0.84	0.214
	Continuity of care	3.00 ± 1.60	2.80 ± 1.32	0.775
	Income	4.13 ± 0.64	3.30 ± 0.82	0.034
	Prestige	3.13 ± 0.83	2.10 ± 0.99	0.033
	Type of diseases	4.50 ± 0.76	4.50 ± 0.85	>0.999
	On calls	3.50 ± 1.41	3.70 ± 1.34	0.762
	Research opportunities	3.38 ± 1.60	3.00 ± 1.56	0.623
	Geographic location	3.88 ± 1.25	3.10 ± 1.37	0.233
Factors influencing Interest in pediatric ophthalmology	I enjoy the pediatric clinic	2.25 ± 1.49	2.90 ± 1.20	0.319
	I like strabismus	3.75 ± 1.75	2.70 ± 1.25	0.157
	Varieties of diseases	2.25 ± 1.39	3.10 ± 1.10	0.166
	Research is very interesting in this field	2.50 ± 1.51	2.30 ± 0.95	0.736
	I enjoy dealing with the pediatric age group	2.00 ± 1.41	3.60 ± 1.26	0.022
	Usually, the outcome is rewarding	3.13 ± 1.46	3.40 ± 1.43	0.693
	Different types of surgeries	3.00 ± 1.69	3.50 ± 1.43	0.507
	Has a good job market	3.75 ± 1.39	3.20 ± 1.32	0.403
Barriers to pediatric ophthalmology	Difficulty dealing with anxious parents	3.63 ± 1.51	3.20 ± 1.32	0.532
	Difficulty examining an uncooperative child	4.88 ± 0.35	3.20 ± 1.32	0.003
	No interest in the subjects of pediatric ophthalmology	3.25 ± 1.75	2.90 ± 1.66	0.671
	Lack of exposure during my training	2.50 ± 1.85	2.10 ± 1.37	0.605
	Poor job market/private income	3.13 ± 0.99	2.50 ± 1.27	0.271
	Covering different subspecialties in pediatric age groups	2.75 ± 1.39	3.10 ± 1.20	0.574

When examining income as a factor influencing career choice, males reported a significantly higher mean score of 4.13 compared to 3.30 for females (p = 0.034). Similarly, prestige showed a significant gender difference, with males rating it higher (mean = 3.13) than females (mean = 2.10, p = 0.033). Other factors, such as type of diseases, on-call duties, research opportunities, and geographic location, did not show statistically significant differences, indicating general agreement between male and female residents on these aspects.

Regarding interest in pediatric ophthalmology, female residents tended to have higher mean scores for enjoyment of pediatric clinics (3.60 vs. 2.00 for males), with this difference reaching statistical significance (p = 0.022). Conversely, males showed higher, though not statistically significant, interest in strabismus (mean = 3.75 vs. 2.70 for females, p = 0.157). Females also rated the variety of diseases slightly higher (mean = 3.10 vs. 2.25 for males), but this difference was not significant (p = 0.166).

When exploring barriers to pursuing pediatric ophthalmology, no significant gender differences were observed for difficulty dealing with anxious parents (males 3.63, females 3.20, p = 0.532). However, males reported significantly greater difficulty examining uncooperative children (mean = 4.88) compared to females (mean = 3.20, p = 0.003). Other barriers, such as lack of interest in the subject, insufficient exposure during training, poor job market or income prospects, and managing multiple pediatric subspecialties, did not differ significantly between genders, suggesting these challenges are perceived similarly by both male and female residents.

## Discussion

This study aimed to investigate the perceptions, attitudes, and factors influencing ophthalmology residents’ career decisions, with a particular focus on the choice of POS subspecialties in Saudi Arabia. The results revealed several key trends among participants, who were mostly in their late 20s and enrolled in residency programs across different regions of the country.

One of the most notable findings was the declining interest in the POS subspecialty, with only 15.3% of residents expressing a preference for this field. The anterior segment and surgical retina subspecialties emerged as the most popular choices, indicating a clear inclination toward more prestigious or adult-focused specialties. Reasons for the low interest in POS were diverse but mainly involved the perceived difficulties in managing anxious parents, examining uncooperative pediatric patients, and a general lack of enthusiasm for the subject matter.

Gender differences were also evident, especially in the factors influencing career choices. Male residents placed greater emphasis on income and prestige, while females showed slightly higher interest in continuity of care and working with pediatric patients, though the latter difference was not statistically significant. These findings highlight the complex interplay of personal and professional factors shaping the career trajectories of ophthalmology residents.

Interpretation of findings

The observed decline in POS subspecialty interest raises important concerns about the future of this field in Saudi Arabia. This trend mirrors global patterns, such as in the United States, where only 7% of ophthalmology residents express interest in pursuing POS training. The challenges associated with pediatric care - dealing with difficult cases and anxious parents - appear to discourage many residents.

Consistent with our findings, studies by Gedde et al. [1] and Alwadani et al. [8] also show a strong preference for anterior segment and surgical retina subspecialties among ophthalmology residents, with notable interest in pediatric ophthalmology and oculoplastics. This pattern suggests that these subspecialties are generally favored, likely due to their perceived prestige, income potential, or job opportunities. Conversely, neuro-ophthalmology and ocular genetics remain less attractive fields, possibly due to fewer perceived opportunities or lower income potential compared to the more popular specialties.

Similarly, De Silva et al.’s study [6] found that specialist registrars showed less interest in pediatrics and strabismus compared to surgical retina and anterior segment, despite pediatric ophthalmology being the third most advertised consultant post.

While our study focuses narrowly on subspecialty preferences with an emphasis on anterior segment and surgical retina, Al-Essa et al.’s research [7] reveals a broader array of career goals. Residents in that study were motivated not only by technical subspecialties but also by the desire to combine medicine and surgery, perform refractive procedures, engage in research, and maintain flexibility for part-time work in the private sector. They also showed a strong preference for urban practice and additional fellowship training, particularly in surgical fields. This more multifaceted perspective suggests the current generation of ophthalmology residents values work-life balance, intellectual challenge, and career customization more than previous cohorts. Understanding these evolving priorities can help tailor recruitment and educational programs to better meet residents’ diverse aspirations.

The gender differences observed add another layer of complexity, suggesting that male and female residents weigh factors differently when making career decisions. Males tend to prioritize income and prestige, whereas females value continuity of care and pediatric patient interaction more, though these differences were not statistically significant. Such gender-based preferences may contribute to uneven specialist distribution across ophthalmology fields, potentially worsening the shortage in POS.

Gedde et al. [1] also note that female physicians across specialties often prioritize work-life balance and patient relationships over financial considerations. Moreover, gender disparities in ophthalmology leadership persist, with women underrepresented among practicing ophthalmologists, faculty, residents, and especially in senior leadership roles such as program directors, department chairs, and society presidents. This “glass ceiling” effect highlights a critical need for efforts to promote gender equity in the field.

Our findings align with Alwadani et al. [8], emphasizing the importance of addressing barriers deterring residents from pursuing POS. Early comprehensive training in pediatric care, support for managing challenging cases, and increased surgical exposure to strabismus could help alleviate concerns. Raising the visibility and prestige of POS subspecialties and developing mentorship programs aimed at younger residents may also foster greater interest. Contrary to common assumptions, financial compensation was not a major factor influencing career choice in POS.

The preference for more popular, less challenging specialties like anterior segment and surgical retina may signal a shift in the ophthalmology workforce, potentially leading to a shortage of POS specialists, already underrepresented in the field. The gender differences further complicate this issue, as they influence how residents prioritize career factors, which could affect specialist distribution.

Solomon et al. [16] advocate for early exposure to neuro-ophthalmology, an often underappreciated subspecialty. Exposure to recently graduated neuro-ophthalmologists across different levels of seniority and practice scope may inspire more residents to consider this career path and rethink its surgical components.

Limitations

Several limitations should be considered when interpreting these results. The cross-sectional study design limits causal inference between influencing factors and actual career choices. Longitudinal studies are needed to track residents’ career paths over time and assess how initial preferences translate into practice.

The reliance on self-reported data introduces potential social desirability bias, where residents may respond in ways they think are expected rather than reflecting their true views. Additionally, restricting the sample to Saudi Arabian ophthalmology residents limits the generalizability of findings to other regions or contexts.

Lastly, external influences such as job market conditions or healthcare policy changes were not explored, yet these may significantly impact career decisions. Future research should incorporate these factors to help achieve a more balanced distribution of specialists, supported by larger, more representative samples.

## Conclusions

This study provides valuable insights into the factors shaping ophthalmology residents’ career choices, particularly highlighting the declining interest in POS subspecialties. The findings underscore the need for targeted interventions to overcome barriers associated with pediatric care and to promote specialization in POS. Addressing these issues may help ensure a more balanced specialist distribution within ophthalmology and meet the growing demand for pediatric eye care in Saudi Arabia. Future research should extend these findings by exploring the long-term impact of residents’ career decisions and the role of external factors. Additionally, understanding how mentorship and early exposure to POS influence interest could offer further strategies to attract residents to this crucial subspecialty.

## Appendices

**Table 12:** Survey template

	Strongly agree	Agree	Neutral	Strongly disagree	Disagree
Challenging diagnostic problems					
Continuity of care					
Income					
Prestige					
Types of diseases					
Oncalls					
Research opportunities					
Geographic location					
Pediatric/strabismus					
Anterior segment					
Medical retina/uveitis					
Surgical retina					
Oculoplastics					
Neuro-ophthalmology					
Glaucoma					
Ocular pathology					
Ocular genetics					
	Strongly agree	Agree	Neutral	Strongly disagree	Disagree
I enjoy pediatric clinic					
I like strabismus					
Varieties of diseases					
Researches are very interesting in this field					
I enjoy dealing with pediatric age group					
Usually, the outcome is rewarding					
Different types of surgeries					
Has a good job market					
	Strongly agree	Agree	Neutral	Strongly disagree	Disagree
Difficulty dealing with anxious parents					
Difficulty examining uncooperative child					
No interests in the subjects of pediatric ophthalmology					
Lack of exposure during my training					
Poor job market/private income					
Covering different subspecialities in pediatric age					
